# Optimal Atmospheric Correction for Above-Ground Forest Biomass Estimation with the ETM+ Remote Sensor

**DOI:** 10.3390/s150818865

**Published:** 2015-07-31

**Authors:** Hieu Cong Nguyen, Jaehoon Jung, Jungbin Lee, Sung-Uk Choi, Suk-Young Hong, Joon Heo

**Affiliations:** 1 Department of Civil and Environmental Engineering, Yonsei University, 134 Shinchon-dong, Seodaemun-gu, Seoul 120-749, Korea; E-Mails: hieunguyen@yonsei.ac.kr (H.C.N.); lionheart_kr@yonsei.ac.kr (J.J.); ortolan@yonsei.ac.kr (J.L.); schoi@yonsei.ac.kr (S.-U.C.); 2 National Academy of Agricultural Science, Rural Development Administration, 166 Nongsaengmyeong-ro, Iseo-myeon, Wanju-gun, Jeollabuk-do 565-851, Korea; E-Mail: syhong67@korea.kr

**Keywords:** *k*-Nearest Neighbor, biomass estimation, DOS, FLAASH, 6S

## Abstract

The reflectance of the Earth’s surface is significantly influenced by atmospheric conditions such as water vapor content and aerosols. Particularly, the absorption and scattering effects become stronger when the target features are non-bright objects, such as in aqueous or vegetated areas. For any remote-sensing approach, atmospheric correction is thus required to minimize those effects and to convert digital number (DN) values to surface reflectance. The main aim of this study was to test the three most popular atmospheric correction models, namely (1) Dark Object Subtraction (DOS); (2) Fast Line-of-sight Atmospheric Analysis of Spectral Hypercubes (FLAASH) and (3) the Second Simulation of Satellite Signal in the Solar Spectrum (6S) and compare them with Top of Atmospheric (TOA) reflectance. By using the *k*-Nearest Neighbor (*k*NN) algorithm, a series of experiments were conducted for above-ground forest biomass (AGB) estimations of the Gongju and Sejong region of South Korea, in order to check the effectiveness of atmospheric correction methods for Landsat ETM+. Overall, in the forest biomass estimation, the 6S model showed the bestRMSE’s, followed by FLAASH, DOS and TOA. In addition, a significant improvement of RMSE by 6S was found with images when the study site had higher total water vapor and temperature levels. Moreover, we also tested the sensitivity of the atmospheric correction methods to each of the Landsat ETM+ bands. The results confirmed that 6S dominates the other methods, especially in the infrared wavelengths covering the pivotal bands for forest applications. Finally, we suggest that the 6S model, integrating water vapor and aerosol optical depth derived from MODIS products, is better suited for AGB estimation based on optical remote-sensing data, especially when using satellite images acquired in the summer during full canopy development.

## 1. Introduction

A forest ecosystem is an important and manageable carbon sink that plays a critical role in reducing carbon concentrations in the atmosphere [1,2,3]. The spatial distribution of above-ground forest biomass (AGB) is necessary for calculating the net flux of terrestrial carbon and supporting climate change modeling studies [4,5,6]. The traditional methods of AGB estimation are based on field sample plots [7,8]. AGB is modeled by using diameter-at-breast-height measurements that are easy to obtain in field samples. Additionally, combining satellite imagery and field inventory offers the distinct advantage that large areas can be monitored [9] and spatial variability can be much better characterized than when using field inventory data exclusively—always assuming that imagery with adequate spatial, spectral and radiometric characteristics is available [10]. However, the quality of satellite imagery ultimately relies on environmental elements including topographic and atmospheric conditions [11]. 

The reflectance of the Earth’s surface is significantly influenced by the atmosphere’s water-vapor and aerosols, which change with time and space. Moreover, the influences of absorption and scattering becomes stronger when target features, such as in aqueous or vegetated areas, are non-bright objects. This problem is especially significant when using optical satellite images of forested areas for monitoring purposes [12]. Therefore, it is crucial to select a reliable and efficient atmospheric correction model among the various available algorithms and software. Also, there are many previous studies that have emphasized the need for atmospheric correction; however, they have stated that it is not necessarily to produce better results for classification and change detection when using single-date data [12,13,14,15,16]. For example, Song *et al.* [16] investigated when and how to correct atmospheric effects on classification and change detection using the maximum likelihood classifier together with a single-date image. Kaufman [17] and Liang *et al.* [18] found that more complicated algorithms provide less accurate classification and change detection results, and thus proposed the relatively simple Dark Object Subtraction (DOS) with and without Rayleigh atmospheric correction. Kawata *et al.* [14] evaluated image classification accuracy before and after atmospheric correction. They used the Lowtran-6 code for removal of atmospheric effects as well as Gaussian maximum likelihood for classification, and concluded that except for the aqueous areas, the atmospheric model did not improve the image classification accuracy. Atmospheric correction’s impact on spectral signatures and vegetation indices, which can lead to uncertainy in AGB estimation, was addressed in [12,19,20]. In those studies, the difference of the mean value of NDVI with and without atmospheric correction was found to be 18%, indicating that atmospheric effects should be removed when using vegetation indices. Lu *et al.* [21] assessed three image-based calibration models (the apparent reflectance model, DOS, and the improved DOS) for biomass estimation in the Amazon Basin. Based on their results, they selected the improved DOS for their further Bio-sphere-Atmosphere Experiment. Uncertainties of information extracted from optical sensor imagery were quantified in [22,23]. These studies revealed that the uncertainties on ocean targets are higher than desert targets due to the lower signal level in aqueous areas. Additionally, in terms of AGB estimation based on remote-sensing data, there are uncertainties and variations of both the spectral values of corrected images and their biomass estimation, which might be the result of atmospheric correction methods [24], shadow and topographic conditions [25,26] or landscape differences [14,23]. However, the impacts of atmospheric correction on above-ground biomass estimation of forested areas have not yet been fully examined.

To date, Landsat images, including Landsat TM and ETM+, have been the most popular medium-resolution data in AGB studies [6,27,28,29,30]. The main reason is that Landsat has been providing Earth observation data the longest, since 1972 [31,32] and also that its spatial and spectral resolutions are in accordance. However, their investigations have been mostly of single scenery, or for single acquisition dates, such as in peak growing season, to establish an AGB model [6]. There has been little research analyzing the differences in seasonal images’ responses to the same National Forest Inventory (NFI) data. Thus, it is worth investigating seasonal Landsat acquisition dates to determine which season is more suitable for AGB estimation and atmospheric correction. 

The main goal of this study was to select an optimal atmospheric correction method for above-ground forest biomass estimation based on remote-sensing data under a certain environmental condition. To achieve this, three of the most popular atmospheric correction models, the DOS, Fast Line-of-sight Atmospheric Analysis of Spectral Hypercubes (FLAASH), and the Second Simulation of Satellite Signal in the Solar Spectrum (6S), were evaluated and compared with Top of Atmospheric (TOA) reflectance. Also, the effectiveness of the atmospheric correction methods for each of the Landsat EMT+ bands under a given atmospheric condition needed to be analyzed in order to determine the dominant method for each band. The test site, the forested Gongju and Sejong regions in South Korea, was chosen, and the evaluation was performed using the *k*-Nearest Neighbor (*k*NN) algorithm with five different seasonal Landsat ETM+ images and field datasets.

## 2. Materials

### 2.1. Study Area

The Gongju and Sejong regions of South Korea was chosen as the study site. They are located in the middle of South Korea (between longitudes 126°53′, 127°25′ E and latitudes 36°17′, 36°43′ N). They have a continental climate, and the forested area covered 72,377 ha in 2010. 

### 2.2. National Forest Inventory Data

**Figure 1:**
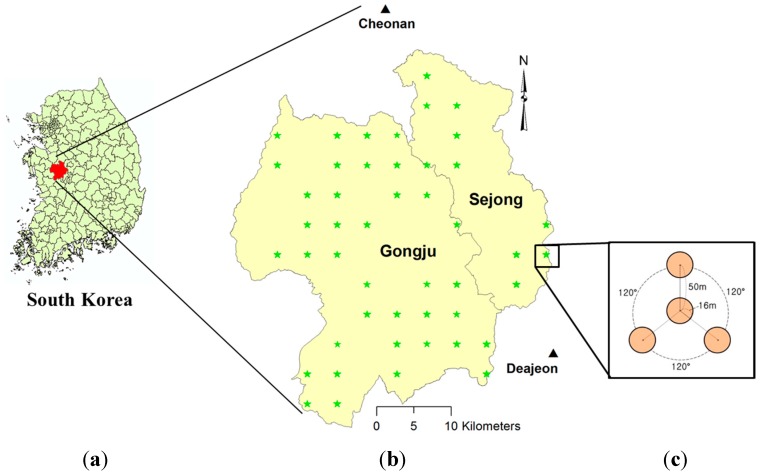
(**a**) Location of the study area, the Gongju and Sejong regions, South Korea; (**b**) locations of field survey (green star) and meteorological stations (black triangle); (**c**) design of NFI plot consisting of 4 sub-plots.

The 5th and 6th NFI data, provided by the Korea Forest Research Institute, were used in this study. It covers 47 plots (144 subplots), and includes location, Diameter at Breast Height (DBH), tree species, age, height, and other data. The AGB of each subplot was estimated from the DBH and tree height according to stem volume models and the biomass expansion factor [33,34,35]. Figure 1 shows the study sites in South Korea along with the distribution of the NFI locations. 

**Table 1:** Atmospheric conditionson date of image acquisition from 2 nearest meteorological stations.

Atmospheric Conditions		Average Temperature (°C)	The Highest Temperature (°C)	The Lowest Temperature (°C)	Relative Humidity (%)	Rainfall (mm)	Total Water Vapor (g/kg)
5 April 2011	Deajeon	10.8	19.5	1.7	30.6	0	2.5
	Cheonan	8.7	18.5	−0.9	36.6	0	2.6
20 May 2011	Deajeon	20.1	24.1	18.4	79.5	13.5	11.8
	Cheonan	20.1	23.9	17.0	79.3	3.0	11.7
8 August 2010	Deajeon	27.9	33.2	23.9	67.6	0	16.2
	Cheonan	27.6	33.6	23.5	73.3	0	17.2
24 October 2009	Deajeon	16.6	23.3	10.0	67.6	0	8.0
	Cheonan	16.1	23.2	10.5	71.5	0	8.2
15 November 2011	Deajeon	6.7	13.0	2.0	57.4	0	3.5
	Cheonan	5.0	12.8	−0.7	65.3	0	3.5

Atmospheric condition information of study site: In order to determine the correlation between AGB estimation and atmospheric condition, the atmospheric conditions of two meteorological stations close to the Gongju and Sejong region were obtained from the Korea Meteorological Administration’s website [36]. These two stations are Deajeon and Cheonan, which are located to the southeast and north of the study site, respectively (Figure 1b). The atmospheric conditions on the date of the Landsat image acquisition from the two stations are summarized in Table 1. It can be seen that the humidity percentage on 20th May 2011 was the highest, followed by 8th August 2010 and 24th October 2011, while it was the lowest on 5th April 2011. There was a rainfall on 20th May 2011, which might have alleviated the relative humidity. The total water vapor at each station for the given dates was calculated based on the average temperature and relative humidity. The total water vapor on 8th August 2010 was the highest, followed by 20th May 2011; the total water vapor levels in the spring (5th April 2011) and late autumn (15th November 2011) were significantly lower. 

### 2.3. Remotely Sensed Data

**Figure 2:**
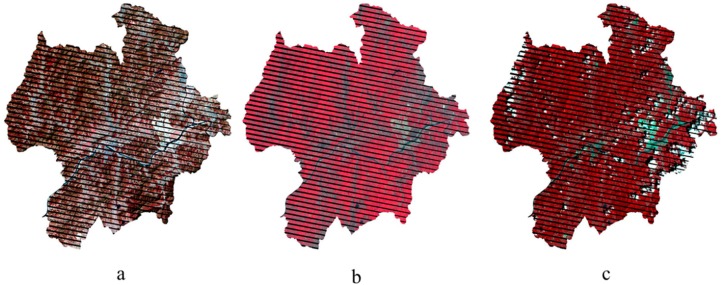

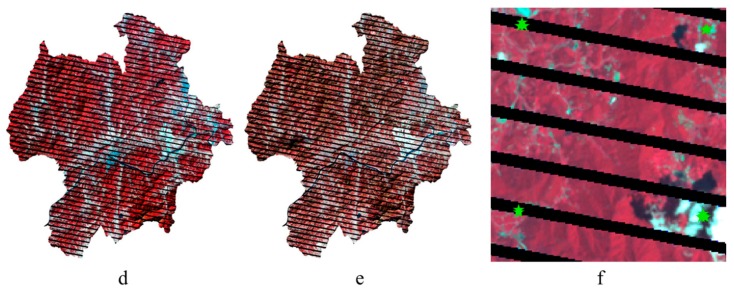
Quality of five seasonal images in infrared composition and study area: (**a**) 4th April 2011; (**b**) 20th May 2011; (**c**) 8th August 2010; (**d**) 24th October 2009; (**e**) 15th November 2011; (**f**) NFI samples from no-data area (black) due to SLC-off ETM+ images, cloud cover or shadow were removed.

The Landsat images for the study were acquired from early spring until late autumn between 2009 and 2011. These dates were 4th April 2011, 20th May 2011, 8th August 2010, 24th October 2009 and 15th November 2011; all five Landsat ETM+ scenes and their information are summarized in Table 2, and are illustrated with the infrared band compositions in Figure 2. They reflected seasonal changes of temperate forest in Korea. The NFI subplots were removed from consideration in cases where they were located in no-data areas due to SLC-off ETM+ images, cloud cover or shadow. 

**Table 2:** Characteristics of Landsat ETM+ images uses in Gongju and Sejong region research.

Scene ID	Date	Path/Row	Season	Sun Azimuth Angle (°)	Sun Elevation Angle (°)
LE71150352011095EDC00	5th April 2011	115/35	Spring	139	53
LE71150352010140EDC01	20th May 2011	115/35	Late spring	124	65
LE71150352010220EDC00	8th August 2010	115/35	Summer	126	61
LE71150352009297EDC00	24th October 2009	115/35	Autumn	155	39
LE71150352011319EDC00	15th November 2011	115/35	Late autumn	159	33

## 3. Methodology

### 3.1. Atmospheric Correction

**Figure 3:**
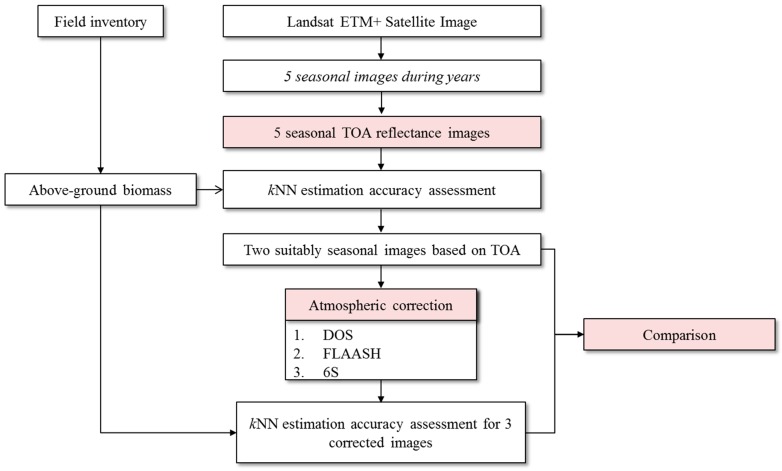
Study flow diagram.

Figure 3 presents an overview of the present study. The biomass estimation procedure consists of seven steps: (1) collection of seasonal satellite images and field survey data (NFI); (2) conversion of digital numbers of all images to top of atmosphere reflectance (TOA); (3) application of *k*NN and ten-fold cross-validation to TOA reflectance images; (4) selection of images with highest accuracy for further study; (5) application of three different atmospheric correction methods to selected images; (6) biomass estimation using *k*NN algorithm and accuracy assessment with 10-fold cross-validation for different atmospheric correction cases, and (7) comparison of all four cases of atmospheric-corrected and non-corrected images. 

Case 1, TOA reflectance: The Landsat ETM+ sensors acquire spectral data and store them in the Digital Number (DN) format (range: 0–255). The original DN value is then converted to TOA reflectance by Equations (1) and (2) [37].

(1) $$L_{\text{λ}}=(\frac{LMAX_{\text{λ}}-LMIN_{\text{λ}}}{Q_{cal\text{max}}-Q_{cal\text{min}}})(Q_{cal}-Q_{cal\text{min}})+LMIN_{\text{λ}}$$

(2) $${\text{ρ}}_{\text{λ}}=\frac{\text{π}L_{\text{λ}}d^{2}}{ESUN_{\text{λ}}\text{cosθ}}$$

where: $L_{\text{λ}}$ is the Radiance at a target band in units of $W/(m^{2}*sr*\text{μ}m)$,

$LMAX_{\text{λ}}$is the Spectral radiance scaled to $Q_{cal\text{max}}$ in units of $W/(m^{2}*sr*\text{μ}m)$,
$LMIN_{\text{λ}}$ is the Spectral radiance scaled to $Q_{cal\text{min}}$ in units of $W/(m^{2}*sr*\text{μ}m)$,
$Q_{cal}$ is the Quantized calibrated pixel value (DN),
$Q_{cal\text{max}}$ is the Maximum quantized calibrated pixel value (DN = 255) corresponding to $LMAX_{\text{λ}}$,
$Q_{cal\text{min}}$ is the Minimum quantized calibrated pixel value (DN = 0) corresponding to $LMIN_{\text{λ}}$,
*d* is the Earth-Sun distance, in astronomical units,
$ESUN_{\text{λ}}$ is the solar irradiance in units of $W/(m^{2}*sr*\text{μ}m)$,
θ is the Sun elevation in degrees (given in satellite image meta data).

Case 2, DOS: This is perhaps the simplest and most widely used image-based absolute atmospheric correction approach [16,25,38,39]. This approach assumes the existence of dark objects throughout a satellite image scene, and they should have zero value, along with a horizontally homogeneous atmosphere (Equation (3)) [25]. Thus, the minimum DN value in the histogram considered as dark objects from the entire scene which is known as the atmospheric effects (mostly from haze), which accordingly is subtracted from all pixels [40]. Several different versions of DOS are currently available in ENVI and ERDAS. In the present study, the water areas having the lowest DN values were chosen as dark objects, in other words the DN values over water areas were set as dark object values:

(3) $$REF=\frac{\text{π}*(L_{\text{λ}}-L_{haze})}{TAU_{v}*(E_{0}*Cos(TZ)*TAU_{z}+E_{down})}$$

where: *REF* is the spectral reflectance of the surface,

$L_{sat}$ is the at-satellite radiance for the given spectral band in units of $W/(m^{2}*sr*\text{μ}m)$,
$L_{haze}$ is the upwelling atmospheric spectral radiance scattered in the direction of and at the sensor entrance pupil and within the sensor’s field of view, in units of $W/(m^{2}*sr*\text{μ}m)$,
$TAU_{v}$ is the atmospheric transmittance along the path from the ground surface to the sensor,
$E_{0}$ is the Solar spectral irradiance on a surface perpendicular to the Sun’s rays outside the atmosphere, in units of $W/(m^{2}*sr*\text{μ}m)$,
*TZ* is the angle of incidence of the direct solar flux onto the Earth’s surface (solar zenith angle),
$TAU_{z}$ is the atmospheric transmittance along the path from the sun to the ground surface,
$E_{down}$ is the down-welling spectral irradiance at the surface due to the scatted solar flux in the atmosphere, in units of $W/(m^{2}*sr*\text{μ}m)$.

Case 3, FLAASH: There is a radiative transfer code named MODerate-Resolution TRANsmittance (MODTRAN4) [41,42], which provides an atmospheric correction software package. FLAASH provides the graphical user interface for the MODTRAN4 spectral calculations, including data simulation [43]. One key feature of FLAASH is that it offers the option of correcting for light scattered into the field of view from adjacent pixels [44]. Basically, it was developed from a standard equation for spectral radiance at a sensor pixel (Equation (4)) [43]:

(4) $$L*=\frac{A\text{ρ}+B{\text{ρ}}_{e}}{1-{\text{ρ}}_{e}S}+L{*}_{a}$$

where: L* is the spectral radiance at a sensor pixel,

ρ is the pixel surface reflectance,
${\text{ρ}}_{e}$ is the average surface reflectance for the pixel and the surrounding region,
*S* is the spherical albedo of the atmosphere,
*A* and *B* are coefficients that vary according to the atmospheric and geometric conditions but not the surface condition,
L*_a_ is the radiance backscattered by the atmosphere.

In MODTRAN4; the *A*, *B*, *S* and L*_a_ parameters are determined by the viewing and solar angles and mean surface elevation. They can vary according to different atmospheric models; aerosol types and visible angles. 

Case 4, 6S: This is also a radiative transfer code, developed from the 5S version [26]. Similarly, 6S was developed from the same Equation (4); however 6S can solve Equation (4) in both scalar and vector form while FLAASH (MODTRAN4) solve the transfer equation only in the scalar form [45]. Thus, the vector 6S version is capable of taking into account light polarization contributions. The requirements for the 6S model are meteorological visibility, type of sensor, sun zenith and azimuth, the date and time of image acquisition, and the latitude and longitude of the scene center. Possible additional input data are atmospheric profiles including water vapor, gas, aerosol, and clouds [24]. In this study, the Atmospheric profiles including the Atmospheric Optical Depth (AOD) and water vapor column were MODIS products: MOD04 [46] and MOD05 [47], respectively. A Matlab routine named “LandCor” was used to control the Fortran code of 6S [48,49].

### 3.2. kNN Estimation

*k*NN is widely utilized in estimation of AGB [50,51,52,53,54]. An unknown pixel is estimated based on the *k*-nearest neighbors that are the most spectrally similar to the target pixel. The spectral distance between a target and reference pixels is calculated as Equation (5) [54] and normalized as Equation (6):

(5) $$d_{t,r}=\sqrt{\sum_{i=1}^{m}{\left(x_{i,t}-x_{i,r}\right)}^{2}}$$

where: *d_t,r_* is the spectral distance between two pixels,

*x_i,t_* is the Reflectance Value (RV) of the target pixel in the *i^th^* band,
*x_i,r_* is the RV of the reference pixel corresponding to a subplot in the *i^th^* band,

and *m* denotes the number of total bands, and:

(6) $$w_{t,r}=\frac{1}{d_{t,r}^{2}}/\sum_{j=1}^{k}\frac{1}{d_{t,j}^{2}}$$

where *j* = 1, 2, …, *r*, *k* indicates the number of nearest neighbors.

Subsequently, the AGB in the target pixel is estimated by summarizing all of the AGB of the *k*-nearest neighbors with respect to their weight, as shown in Equation (7):

(7) $${\hat{y}}_{t}=\sum_{r=1}^{k}\left(w_{t,r}\times y_{r}\right)$$

where *ŷ_t_* is the AGB at target pixel *t*, and *y_r_* is the AGB value of the *k*-nearest subplots.

### 3.3. Accuracy Assessment: 10-Fold Cross-Validation

To quantify the RMSE, all field plots are divided into 10 equal-sized subsets. In validation, 9 subsets are used for calibration, and the remaining one is used for validation. The RMSE is calculated as Equation (8) [55]:

(8) $$\text{RMSE}=\sqrt{\frac{1}{n}\sum_{i=1}^{n}{\left({\hat{y}}_{i}-y_{i}\right)}^{2}}$$

where *ŷ_i_* is the estimated AGB of the *i^th^* observation, *y_i_* is the AGB from the reference dataset, and *n* is the number of subplots.

This process is repeated in ten times, and then the mean RMSE finally is obtained. Additionally, the relative RMSE is calculated as in Equation (9):

(9) $$\text{RMSE\%}=\frac{\text{RMSE}}{\bar{y}}\times 100\%$$

where the RMSE is calculated by Equation (8), and $\bar{y}$ is the observed mean.

### 3.4. Optimal Atmospheric Correction Method for Particular Band

**Figure 4:**
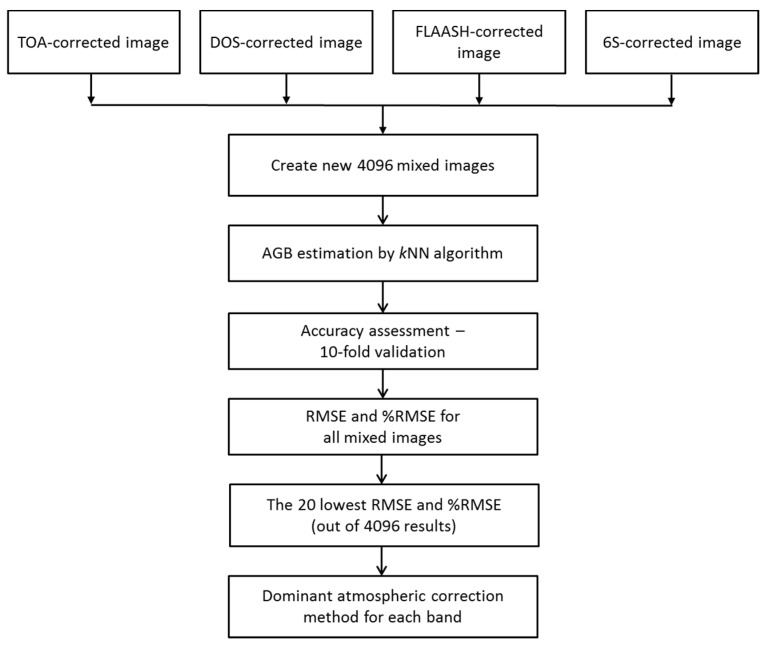
Matlab routine for finding dominant atmospheric correction method for each band.

Figure 4 shows the proposed routine to test whether a particular atmospheric correction method is more suitable for a particular band of a specific seasonal Landsat ETM+ image. For the test, all four corrected Landsat images (TOA, DOS-corrected, FLAASH and 6S-corrected) were mixed together, and then 4096 (=4^6^) combinations from six bands with different atmospheric correction methods were produced. With respect to all 4096 combinations, the *k*NN algorithm and accuracy assessment were performed, and consequently, 4096 accuracy assessment results were produced. The top 20 lowest RMSE from those were selected for the analysis of the dominance of the atmospheric correction method on each band. In this manner, the advantages of each atmospheric correction method for a particular Landsat ETM+ band and a given specific atmospheric condition were examined and analyzed.

## 4. Results and Discussion

In this study, we performed experiments on the test site, which was the forested area of Gongju and Sejong region, South Korea, using five seasonal Landsat ETM+ images. The experiments are summarized in Figure 3 and Figure 4. First, we applied the *k*NN algorithm to the TOA reflectance values of all five images to confirm the most suitable season for AGB estimation. Second, we applied the three atmospheric correction methods (DOS, FLAASH, 6S) to the suitable ETM+ images selected from the first test—8th August 2010 and 20th May 2011 and applied the *k*NN algorithm and accuracy assessment to determine the best method for AGB from the perspective of estimation accuracy. Third, the optimal correction methods for each ETM+ image band were investigated as indicated in Figure 4. The experimental results are discussed in the following paragraphs. 

### 4.1. Seasonal AGB Estimation

**Figure 5:**
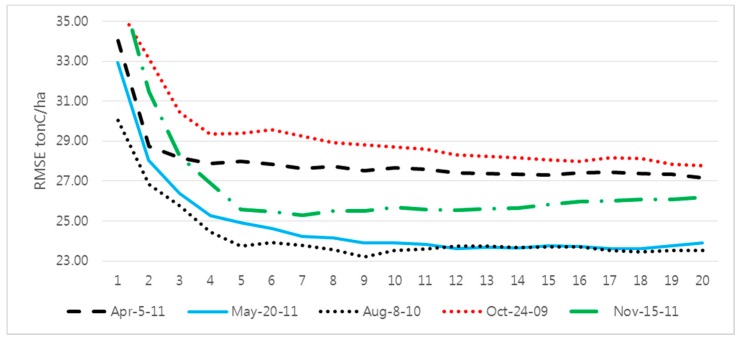
Changing of RMSE due to increase of *k*.

Table 3 shows the accuracy assessment results in the RMSE and relative RMSE of the AGB estimation with five ETM+ images and NFI field data. The number of *k*-nearest neighbors from 1 to 20 was tested, and Figure 5 illustrates the pattern of RMSE changes while *k* is changing with respect to five different seasonal images. Overall, when *k* is more than 6, RMSE stays more stable, and the variation of relative RMSE that has *k* between 6 and 20 is less than 1.3% in all cases except for the autumn image (the variation was 3.6%). The accuracy levels also show agreement with an earlier AGB estimation for the Danyang area [56] and another, previous study in the same area [57].

**Table 3:** Accuracy assessment results of AGB at Gongju and Sejongsites in different seasons: RMSE (unit: tonC/ha) and %RMSE (unit: %).

Date	5 April 2011		20 May 2011		8 August 2010		24 October 2009		15 November 2011	
Season	Spring		Late Spring		Summer		Autumn		Late Autumn	
*k*	RMSE	%RMSE	RMSE	%RMSE	RMSE	%RMSE	RMSE	%RMSE	RMSE	%RMSE
1	34.0	63.6	32.9	63.0	30.1	58.2	35.8	69.5	37.2	73.8
2	28.8	53.7	28.1	53.6	26.8	52.0	33.2	64.4	31.5	62.5
3	28.2	52.6	26.4	50.4	25.8	49.9	30.5	59.2	28.3	56.1
4	27.9	52.1	25.3	48.3	24.5	47.4	29.4	57.0	26.9	53.4
5	28.0	52.3	24.9	47.6	23.8	46.0	29.4	57.1	25.6	50.8
6	27.9	52.0	24.6	47.1	23.9	46.3	29.6	57.5	25.5	50.6
7	27.7	51.6	24.2	46.4	23.8	46.1	29.3	56.8	25.3	50.2
8	27.7	51.8	24.1	46.2	23.6	45.6	28.9	56.2	25.5	50.6
9	27.5	51.4	23.9	45.7	23.2	45.0	28.8	56.0	25.5	50.7
10	27.7	51.7	23.9	45.7	23.5	45.6	28.7	55.8	25.7	51.0
11	27.6	51.5	23.8	45.6	23.6	45.8	28.6	55.6	25.6	50.7
12	27.4	51.2	23.6	45.1	23.7	46.0	28.3	55.0	25.6	50.7
13	27.4	51.2	23.7	45.3	23.8	46.0	28.3	54.9	25.6	50.9
14	27.3	51.1	23.6	45.2	23.7	45.9	28.2	54.8	25.6	50.9
15	27.3	51.0	23.8	45.4	23.7	46.0	28.1	54.5	25.8	51.3
16	27.4	51.2	23.7	45.4	23.7	46.0	28.0	54.4	26.0	51.6
17	27.5	51.3	23.6	45.2	23.5	45.6	28.2	54.8	26.0	51.6
18	27.4	51.1	23.6	45.2	23.5	45.5	28.2	54.7	26.1	51.8
19	27.3	51.0	23.8	45.4	23.5	45.6	27.8	54.1	26.1	51.8
20	27.2	50.7	23.9	45.7	23.5	45.6	27.8	53.9	26.2	52.0

Regarding the seasonal/temporal pattern of AGB estimation, the best RMSE was achieved when using the 8 August 2011 image, followed by 20 May 2011 (Figure 5). The other AGB estimations, during spring and autumn, presented less accurate results. The results show agreement with an assessment of AGB estimation with a Landsat time series in southeast Ohio, which is at a similar latitude in the northern hemisphere and has a similar seasonal pattern to that of the study site [6]. They reported that the summer period can be suitable time to estimate AGB using Landsat time-series data. Our results also confirmed that a forest in full canopy development presents better results than its early development around the late spring or in the defoliating season. 

### 4.2. Comparison of Atmospheric Correction Methods for AGB

The two Landsat ETM+ images on 20th May 2011 and 8th August 2010, which had the lowest RMSE of AGB estimation, were chosen to compare the performance of three atmospheric correction methods: DOS, FLAASH, and 6S. First, Table 4 shows that in the AGB estimation accuracies for 20th May 2011 with four models (the just-noted three plus TOA reflectance), there is a common trend in that at each *k*, 6S consistently showed better results than the others; and even the FLAASH results showed a little improvement over TOA and DOS. The best RMSE was achieved by the 6S-corrected image with *k* = 6 and *k* = 8: its RMSE and relative RMSE were 22.5 tonC/ha and 43.1%, respectively, and the improvement of the relative RMSE was around 4% and 3.1% compared with the TOA cases (47.1% and 46.2%) at *k* of 6 and 8, respectively. Second, Table 5 shows the AGB estimation accuracies of 8th August 2010 with the same four models. Similarly, the 6S model showed better improvement at each *k* when FLAASH does not improve, and the DOS results are about the same as 6S. Additionally, the magnitude of improvement by 6S in the 8th August 2010 image was greater than the improvement by 6S in the 20th May 2011 image. The best RMSE and relative RMSE of the 20th May 2011 image corrected by 6S were 21.3 tonC/ha and 41.3%, respectively, at *k* = 6; this represented an improvement of 5% relative RMSE over the TOA case (46.3). 

**Table 4:** Accuracy assessment results for 20th May 2011 Landsat ETM+ by four atmospheric cases: RMSE (unit: tonC/ha) and %RMSE (unit: %).

*k*	TOA Reflectance		DOS		FLAASH		6S	
	RMSE	%RMSE	RMSE	%RMSE	RMSE	%RMSE	RMSE	%RMSE
1	32.9	63.0	32.9	63.0	29.1	55.6	28.8	55.1
2	28.1	53.6	28.1	53.6	26.7	51.1	24.9	47.6
3	26.4	50.4	26.4	50.4	24.5	46.8	23.5	44.9
4	25.3	48.3	25.3	48.3	24.5	46.9	23.0	44.0
5	24.9	47.6	24.9	47.6	24.4	46.7	22.9	43.7
6	24.6	47.1	24.6	47.1	24.1	46.1	22.5	43.1
7	24.2	46.4	24.2	46.4	24.0	45.9	22.9	43.7
8	24.1	46.2	24.1	46.2	24.0	45.8	22.5	43.1
9	23.9	45.7	23.9	45.7	24.1	46.0	22.7	43.4
10	23.9	45.7	23.9	45.7	23.8	45.6	22.9	43.7
11	23.8	45.6	23.8	45.6	23.7	45.3	22.9	43.8
12	23.6	45.1	23.6	45.1	23.5	44.9	22.7	43.3
13	23.7	45.3	23.7	45.3	23.5	45.0	22.7	43.5
14	23.6	45.2	23.6	45.2	23.7	45.3	22.9	43.9
15	23.8	45.4	23.8	45.4	23.7	45.4	23.1	44.1
16	23.7	45.4	23.7	45.4	23.6	45.2	23.3	44.6
17	23.6	45.2	23.6	45.2	23.6	45.2	23.4	44.8
18	23.6	45.2	23.6	45.2	23.7	45.4	23.4	44.8
19	23.8	45.4	23.8	45.4	23.8	45.5	23.5	44.9
20	23.9	45.7	23.9	45.7	23.8	45.5	23.4	44.7

**Table 5:** Accuracy assessment results for 8th August 2010 Landsat ETM+ by four atmospheric cases: RMSE (unit: tonC/ha) and %RMSE (unit: %).

*k*	TOA Reflectance		DOS		FLAASH		6S	
	RMSE	%RMSE	RMSE	%RMSE	RMSE	%RMSE	RMSE	%RMSE
1	30.1	58.2	30.1	58.2	31.0	60.1	24.9	48.3
2	26.8	52.0	26.8	52.0	27.6	53.4	23.4	45.3
3	25.8	49.9	25.8	49.9	25.4	49.3	23.2	44.9
4	24.5	47.4	24.5	47.4	24.3	47.1	21.9	42.3
5	23.8	46.0	23.8	46.0	23.8	46.0	21.5	41.7
6	23.9	46.3	23.9	46.3	23.8	46.1	21.3	41.3
7	23.8	46.1	23.8	46.1	23.8	46.1	21.8	42.3
8	23.6	45.6	23.6	45.6	23.5	45.5	21.6	41.9
9	23.2	45.0	23.2	45.0	23.5	45.6	21.5	41.6
10	23.5	45.6	23.5	45.6	23.7	46.0	21.4	41.4
11	23.6	45.8	23.6	45.8	23.7	45.9	21.5	41.7
12	23.7	46.0	23.7	46.0	23.9	46.3	21.7	42.0
13	23.8	46.0	23.8	46.0	23.8	46.0	21.8	42.2
14	23.7	45.9	23.7	45.9	23.6	45.7	21.8	42.2
15	23.7	46.0	23.7	46.0	23.7	46.0	22.0	42.6
16	23.7	46.0	23.7	46.0	23.7	45.9	22.1	42.7
17	23.5	45.6	23.5	45.6	23.7	45.9	22.1	42.8
18	23.5	45.5	23.5	45.5	23.8	46.0	22.1	42.9
19	23.5	45.6	23.5	45.6	23.7	46.0	22.2	43.1
20	23.5	45.6	23.5	45.6	23.7	46.0	22.4	43.4

**Figure 6:**
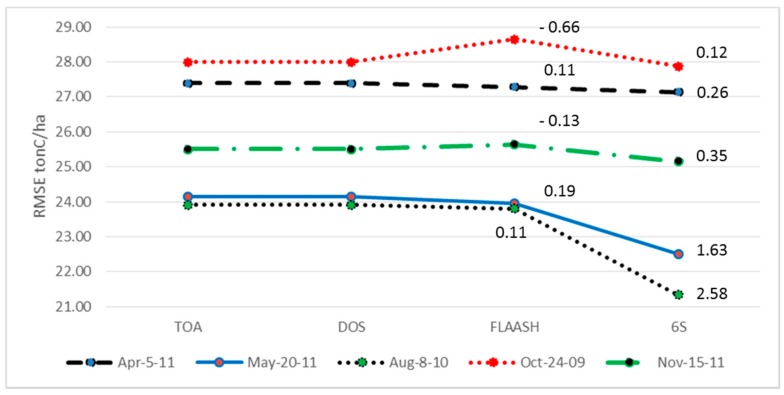
RMSE of AGB estimation with application of atmospheric correction methods at Gongju and Sejong region. The numbers in the graph are the RMSE differences between FLAASH and TOA and between 6S and TOA (the difference between DOS and TOA was ignored).

Figure 6 summarizes the results with the lowest RMSE for all of the seasonal images with the different atmospheric correction methods. In each seasonal image, the lowest RMSEs were achieved with the image corrected by the 6S method at the same *k*, and then the RMSEs of the three other cases (TOA, DOS and FLAASH) at the same *k* were chosen for comparison. The *k* values for 5th April 2011, 8th August 2010, 24th October 2009 and 15th November 2011 were 13, 6, 16 and 8, respectively. In the case of 20th May 2011, 6S’s RMSE attained the lowest point at *k* = 6 and 8, as noted in the previous section; however, at *k* = 8, TOA’s RMSE was still maintaining a decreasing trend; thus *k* = 8 was chosen to add to Figure 6. This figure confirms that the 6S method performed better than the others, and that the late spring and summer images provided the lower RMSE for AGB estimation; contrastingly, FLAASH’s results were inconsistent in all seasons, and DOS did not improve the RMSE of AGB estimation. Additionally, 6S showed a significant improvement compared to TOA in the case of atmospheric conditions in which the total water vapor peaked at the highest values (Table 1), while the 6S result improved only marginally compared with TOA when the total water vapor was not high, such as in Korea’s early spring. Shaw [58] proved that air masses at high temperatures contain more aerosols than at low temperatures. In this regard, we found a correlation between temperature and total water vapor: in AGB’s RMSE result (Table 1 and Figure 6), the trend shown was at higher temperatures there were more aerosols and total water vapor, under which condition, AGB estimation by the 6S model was more improved. For example, the largest reduction of RMSE on 8th August 2010, around 2.6 tonC/ha, was by 6S (Figure 6) when the temperature and the total water vapor were both the highest, followed by 20th May 2011.

These AGB results showed agreement with Nazeeretal. [59], who found that 6S presented the lowest difference between the surface reflectance measured from fields and the response Landsat ETM+ values. On the basis of the above experiments for AGB estimation and accuracy assessment, it can be claimed that atmospheric correction by 6Sis more advantageous, particularly AGB estimation, than by the other methods, especially with ETM+ images of full canopy under atmospheric conditions characterized by high total water vapor and high temperature, and consequently high aerosol levels. 

### 4.3. Optimal Atmospheric Correction Method for a Particular Band

The process described in Figure 4 was applied for two Landsat ETM+ scenes (20th May 2011 and 8th August 2010). However, choosing an optimal number of nearest neighbors (*k*) is a crucial point with respect to the use of the *k*NN algorithm [54]. In the present study, to select the optimal *k* between 1 and 20, RMSE results from five seasonal Landsat ETM+ were tested in the previous section (Table 3 and Figure 5). There was a common trend, which was that with increasing number of *k*, the RMSEs normally decreased, and became more stable after the value of *k* = 6. Nevertheless, the minimum RMSEs could be obtained at different *k* based on different seasonal images; hence the proposed routine used *k* at 6 for the *k*NN algorithm for all new images, given that these RMSEs were very close to those at larger *k*, and that the larger *k* values could average out the variation of the original sample plot data in the pixel-level estimates [10,52,54,60]. Basically, 4096 combinations of images, including six bands in each image, were created, and the *k*NN algorithm was run to calculate the AGB RMSE and relative RMSE. In each image, six bands were successively chosen from four corrected images for the same acquisition date. Then, all of the RMSE and relative RMSE of all of the images were listed and compared with each other. Table 6 and Table 7 show the top 20 lowest RMSE and relative RMSE for 20th May 2011 and 8th August 2010, respectively. 

As can be seen, there was significant improvement in the RMSE of AGB estimation when using combinations for 20th May (late spring), whereas there was a small decrease in the RMSE for 8th August (the summer, when the canopy is in full development). In the case of 20th May 2011, the RMSE decreased from 22.5 tonC/ha and 43.1.1% with only 6S correction at *k* = 6, to 19.5 tonC/ha and 37.2% RMSE with the following combination (an improvement of 6% relative RMSE): bands 1 and 6 corrected by FLAASH; bands 2, 4 and 5 corrected by 6S, and band 3 corrected by TOA. Regarding 8th August 2010, the best combination was the following: band 1 corrected by FLAASH; bands 2, 3, 4 and 5 corrected by 6S, and band 6 corrected by DOS. The improvement by combining was not so remarkable compared with the 6S correction image (21.3 tonC/ha, 43.1% in case of 6S correction and 21.2 tonC/ha, 41.1% in case of combination).In both cases, 6S overall showed a better performance than the other three models: TOA, DOS and FLAASH. Although there was no consistently best combination of atmospheric correction methods, one pattern was recognized: 6S performed the best for band 4 (0.76–0.90 µm) and band 5 (1.55–1.75 µm) of ETM+, which was most affected by absorption of water vapor, well-illustrated in the atmospheric window, and which bands are the pivotal ones for biomass content estimation and vegetation applications [61]. This result is further supporting evidence that 6S is more advantageous than the other methods. 

**Table 6:** Accuracy assessment results for 20th May 2011 Landsat ETM+ by 4 atmospheric cases: RMSE (unit: tonC/ha) and %RMSE (unit: %); for band area: 4 atmospheric correction options, TOA, DOS, FLAASH and 6S are named 1, 2, 3 and 4, respectively.

RMSE	%RMSE	Band 1	Band 2	Band 3	Band 4	Band 5	Band 6
19.5	37.2	3	4	1	4	4	3
19.5	37.2	3	4	2	4	4	3
19.8	38.0	1	4	3	4	4	3
19.8	38.0	2	4	3	4	4	3
19.9	38.0	3	4	3	4	4	3
19.9	38.0	4	4	1	4	4	3
19.9	38.0	4	4	2	4	4	3
20.1	38.5	1	3	1	4	4	3
20.1	38.5	2	3	1	4	4	3
20.1	38.5	1	3	2	4	4	3
20.1	38.5	2	3	2	4	4	3
20.2	38.6	4	4	3	4	4	3
20.2	38.7	4	2	1	4	4	3
20.2	38.7	4	1	1	4	4	3
20.2	38.7	4	2	2	4	4	3
20.2	38.7	4	1	2	4	4	3
20.2	38.7	3	3	1	4	4	3
20.2	38.7	3	3	2	4	4	3
20.3	38.7	1	2	1	4	4	3
20.3	38.7	1	1	1	4	4	3
Optimal method		3, 4, 2, 1	4, 3, 2, 1	1, 2, 3	4	4	3

**Table 7:** Accuracy assessment results for 8th August 2010 Landsat ETM+ by four atmospheric cases: RMSE (unit: tonC/ha) and %RMSE (unit: %); for band area: four atmospheric correction options, TOA, DOS, FLAASH and 6S are named 1, 2, 3 and 4, respectively.

RMSE	%RMSE	Band 1	Band 2	Band 3	Band 4	Band 5	Band 6
21.2	41.1	3	4	4	4	4	2
21.2	41.1	3	4	4	4	4	1
21.2	41.2	1	4	4	4	4	3
21.2	41.2	2	4	4	4	4	3
21.3	41.2	4	4	4	4	4	2
21.3	41.2	4	4	4	4	4	1
21.3	41.2	3	4	4	4	4	3
21.3	41.2	3	4	4	4	4	4
21.3	41.3	1	4	4	4	4	2
21.3	41.3	2	4	4	4	4	2
21.3	41.3	1	4	4	4	4	1
21.3	41.3	2	4	4	4	4	1
21.3	41.3	4	4	3	4	4	3
21.3	41.3	4	4	4	4	4	4
21.3	41.4	1	4	4	4	4	4
21.3	41.4	2	4	4	4	4	4
21.4	41.4	4	4	4	4	4	3
21.4	41.4	3	4	2	4	4	3
21.4	41.4	3	4	1	4	4	3
21.4	41.5	4	4	3	4	4	2
Optimal method		3, 1, 2, 4	4	4	4	4	2, 1, 3, 4

### 4.4. Atmospheric Correction Method for Above-Ground Forest Biomass Estimation

In summation, 6S (with LandCor interface), when compared with TOA, DOS and FLAASH, was decisively the most effective method for AGB estimation with the *k*NN algorithm using ETM+; 6S performed especially well in band 4 and band 5 (the infrared wavelengths). Those bands have been found to be valuable for assessment of plant chlorophyll concentration [62] and LAI [63], both of which are closely correlated with AGB [64]. 

The present results could have been due to the specific atmospheric parameters involved in each of the models. While DOS is not concerned with atmospheric profile [25], and FLAASH uses global values for its atmospheric parameters [41,42], 6S exploits the MODIS products, MOD04 [46] and MOD05 [47] (representing total water vapor and aerosol optical depth). In more detail, the atmospheric component consists of water vapor, aerosol, ozone, oxygen, carbon dioxide, and nitrogen, which apparently influence path radiance [26]. However, the influence level of each constituent differ; total ozone, total water vapor and aerosol optical depth are considered the key atmospheric constituents [49]. Whereas total ozone is assumed not to vary with ground elevation across a Landsat scene, and is located mostly in the stratosphere [65], total water vapor and aerosol optical depth vary by ground elevation, since most of their constituents are located in the lower troposphere [26]. Zelazowski *et al*. [49] stated that total ozone has minor, diminishing effects on the spectral signature, mainly in the range of Landsat’s band 2, whereas by contrast, water vapor and aerosol can have major influences on the signal registered by all Landsat bands. Furthermore, the present study site experiences high total water vapor and high temperature during the summer season [36], and thus it is also effected by aerosol optical depth [58,66]. Additionally, from the accuracy assessment results (Figure 5 and Figure 6) in comparison with Table 1, it was confirmed that Landsat ETM+ acquired in the full canopy season, when both temperature and total water vapor are at high levels, provides the most accurate RMSE. Thus, under such atmospheric conditions, the 6S model, with water vapor and aerosol optical depth inputs from the MODIS products (MOD04 and MOD05), offers the advantage in AGB estimation.

## 5. Conclusions

In this study, Landsat ETM+ imagery and NFI field survey data were used to evaluate the accuracy of above-ground biomass estimation using the *k*NN algorithm with the three atmospheric correction methods DOS, FLAASH, and 6S. The evaluations were conducted for a forested area in the Gongju and Sejong regions, South Korea, using images acquired from early spring to late autumn. From a comparison of those atmospheric correction cases and seasonal images over the study area, it was found that the lowest RMSE of the AGB was achieved when using the 6S radiative transfer code. The second highest accuracy was achieved in the FLAASH-corrected images, the AGB results from the DOS-corrected images being almost the same as those from the TOA-corrected results. Also, this study reconfirmed that satellite images with full canopy are the best for AGB estimation.

A practical method of finding an optimal combination of atmospheric correction methods for each band was suggested and tested. Although the combination was dominated by 6S, it was shown that a combination of different atmospheric correction methods could contribute considerably to improving AGB accuracy. Furthermore, in the AGB results for the mixing of atmospheric correction methods, there was a consistency in that 6S performed much better than the others in bands 4 and 5. It can be speculated that the superior performance reflected the fact that 6S brings in atmospheric parameters including total water vapor and aerosol optical depth from the MODIS products. 

It is understood that correction of the atmospheric effect generally requires a series of complex steps; however, this is necessary not only specifically for AGB applications but also for forestry applications in general. There remains a need for further comparison among the different atmospheric correction methods in order to determine the optimal methods under certain atmospheric conditions. However, from the results obtained in the present study, presented above, we can at least suggest that the 6S model, integrating water vapor and aerosol optical depth derived from the MODIS products, is better suited for AGB estimation based on optical remote-sensing data, especially when using data that are acquired in summer, when total water vapor and temperature are both high and the forest canopy is in full development.

## Abbreviations

*k*NN: *k*-Nearest Neighbor
DOS: Dark Object Subtraction
FLAASH: Fast Line-of-sight Atmospheric Analysis of Spectral Hypercubes
6S: the Second Simulation of Satellite Signal in the Solar Spectrum
